# Post-saccadic Eye Movement Indices Under Cognitive Load: A Path Analysis to Determine Visual Performance

**DOI:** 10.18502/jovr.v17i3.11578

**Published:** 2022-08-15

**Authors:** Marzieh Salehi Fadardi, Javad Salehi Fadardi, Monireh Mahjoob, Hassan Doosti

**Affiliations:** ^1^Department of Optometry & Vision Sciences, University of Melbourne, Melbourne, Australia; ^2^Department of Psychology, Ferdowsi University, Mashhad, Iran; ^3^Claremont Graduate University, California, USA; ^4^School of Psychology, Bangor University, Bangor, UK; ^5^Health Promotion Research Center, Department of Optometry, Rehabilitation Faculty, Zahedan University of Medical Sciences, Zahedan, Iran; ^6^Department of Statistics, Macquarie University, Sydney, Australia

**Keywords:** Eye Movement, Saccades, Task Performance

## Abstract

**Purpose:**

The evidence on the linear relationship between cognitive load, saccade, fixation, and task performance was uncertain. We tested pathway models for degraded task performance resulting from changes in saccadic and post-saccadic fixation under cognitive load.

**Methods:**

Participants' (*n* = 38) eye movements were recorded using a post-saccadic discrimination task with and without arithmetic operations to impose cognitive load, validated through recording heart rate variability and subjective measurement.

**Results:**

Results showed that cognitive load led to longer latencies of saccade and fixation; more inaccurate responses and fewer secondary saccades (*P* < 0.001). Longer saccade latencies influenced task performance indirectly via increases in fixation latency, therefore, longer reaction times and higher response errors were observed due to limited fixation duration on desired target.

**Conclusion:**

We suggest that latency and duration of fixation indicate efficiency of information processing and can predict the speed and accuracy of task performance under cognitive load.

##  INTRODUCTION

Saccadic eye movements are rapid and conjugate eye movements that voluntarily move the eyes from one target to another. Several parts of the cerebral cortex, such as the frontal eye field and the parietal eye field, play an important role in performing a saccade.^[1]^

An individual's performance of visual tasks can be degraded due to task conditions such as cognitive load and mental stress.^[2,3,4,5,6,7,8]^ Cognitive load refers to the amount of information that working memory deals with at a time. Visual tasks under high cognitive load conditions can limit the application of top–down processes and result in poor task performance.^[2,3,4,5,6,7,8]^ Distracted minds can also lead to the degradation of an individual's performance during daily activities where both accuracy and speed are required to deal with visual targets under high load situations such as driving. In order to see a target, individuals require both to get the eyes on the desired target (fixation) that is to “collect” the sensory visual inputs-, and then to “process” the consequent visual information. However, this is not always the case, even for normally sighted individuals. In some instances, it has been reported that individuals have looked at but failed to see a visual target or saw it very late while driving and doing another task at the same time.^[9]^

Previous investigations in normally sighted individuals have reported that cognitive demands of concurrent tasks can affect saccade eye movements and individuals' visual task performance.^[2,3,4,5,6,7]^ Fadardi and Abel found that both latencies of saccade and fixation on the desired target increased and task performance decreased when participants were required to perform a post-saccadic task concurrently with an arithmetic task.^[8]^ They suggested that such changes in saccades may give rise to late eye fixation which itself may further limit fixation duration and efficiency of information processing under time-restricted situations.^[8]^ However, the cause–effect relationship between the changes in saccades and post-saccadic task performance is not yet clear.

Path analysis is an extension of multiple regression statistical analysis that is used to evaluate causal models by examining the relationships between several dependent variables and independent variables. This method as a methodological tool can estimate the magnitude and strength of effects in causal connections between variables. In addition, path analysis is useful for comparing different causal models to examine the best fit with the data.^[10]^

Here, we aim to investigate the cause–effect relationship between changes in saccade characteristics, fixation duration, accuracy, and speed of task performance within subjects. We have hypothesized that task-induced changes in saccades give rise to the degradation of an individuals' performance of post-saccadic tasks indirectly by imposing changes in eye fixation on the desired target. The results of this study have agreed with this theory and has also suggested that the timing aspects of fixation (latency and duration) which may give rise to poor visual task performance may also be utilized in its prediction.

##  METHODS

### Participants

Thirty-two graduate students from the University of Melbourne (24 females and 8 males) with a mean age of 30.81 ± 12.5 years participated in the experiment. All participants had normal general health condition and a visual acuity ≥ 6/9 in each eye. Participants were requested to avoid sedatives and alcohol consumption the night before the tasks.

### Apparatus and Procedures

All procedures contributing to the current study conformed to the Declaration of Helsinki and were approved by the institutional review board of the University of Melbourne. All participants volunteered for the experiment, and the signed written informed consent was obtained from them.

The tasks were presented using the SR Research Experiment Builder 1.6.2 (SR Research, Mississagua, Ontario) and a 1024 × 728 NEC-WT610 projector with a screen placed at a distance of 160 cm from participants. Participants were required to look at a fixation point presented at the central gaze position until it disappeared within a random time between 1500—3000 ms. The fixation point was followed by a Tumbling E target presented randomly across ± 25º horizontally away from center in 5º steps. Participants were required to look at and promptly discriminate the direction of the E target (right or left) using a game player console. Participants were highly encouraged to do the tasks with the highest accuracy and speed as much as possible. The submission of manual responses was followed by the fixation target re-appearing and participants were required to look back to the center and wait for another E target to be presented. The size of the Tumbling E and fixation targets were the same; that is, 0.3 Log MAR. Each eccentric gaze position was tested every five times.

The task was repeated with a concurrent arithmetic task to impose a high level of mental load within participants. For the arithmetic task, the participants were required to continuously subtract 7 from a number between 100 and 200 and verbalize the arithmetic responses in 0.5 s. Participants' performance on the arithmetic task was recorded and was qualitatively monitored after the experiment was terminated. Task performance was measured using participants' reaction time to the E targets and the percent rate of accurate responses was manually submitted. The time-related variables including participants' reaction time and latency of eye movements were measured using the onset time of each target on the screen; that was determined by installing a photocell in a corner of the screen which recorded luminance changes.

Eye movements were recorded during the tasks using an EyeLink II high-speed head mounted video eye tracker (SR Research, Mississagua, Ontario) at a sampling rate of 500 Hz in pupil tracking mode with an accuracy of 0.5º or better. Head movements were minimized during the tasks using an adjustable supportive headrest. Participants' head movements were also qualitatively evaluated offline by plotting the positions of the head markers of the EyeLink II.

Concurrent heart rate recordings and a retrospective subjective rating score using a computer version of NASA TLX (Task Load Index) were used to confirm within-subjects' variation in level of cognitive load across the task conditions.

### Eye Movements Analysis

Using the analogue output from the EyeLink II (resampled at 1000 Hz) allowed us to synchronize the time of the eye movements with the target timing. The digital output from EyeLink II was used for the analysis of eye position in MATLAB R2012a after being digitally low-pass filtered with a cut-off at 70Hz. A bidirectional filter (using the Matlab filtfilt command) was applied to the data to avoid time delays. The filter used a mean squares algorithm to produce the least error signal; that is, the difference between the desired and the actual signal. Velocity data were derived from the position data and smoothed using a second-order differentiator with a cut-off frequency of 62.5 Hz.

Saccades were selected using a custom written MATLAB program that presented potential saccades for manual review and selection. The program used the criteria of 10º/s and 8000º/s^[2]^ for saccade onset and offset after the target step. Trials associated with blinks, predictive saccades, or saccades with directional error or gain of < 0.5 or > 1.5 were not included in the analysis.^[11]^ Asymptotic peak velocity, saccade latency, and gain were measured under each task condition. Occurrence of any secondary saccade was also marked for each trial.

Latency of initial eye fixation, also termed target acquisition time,^[8]^ was determined as the time between the target onset on the screen and the initial placement of the eye gaze within 0.5º of the target of interest which was typically located at the end of the saccadic eye movements. Trials with fixation losses due to saccades larger than 1º were not included in the analysis. Fixation duration was measured as the time of duration of fixating on the target before a manual response to the target of either right or left, was submitted.

### Data Analysis

Task conditions were compared within participants to determine whether any significant effects on task performance exist, and to monitor saccade eye movements; that is, latency, gain, and velocity of pre-saccades, the probability of the occurrence of secondary saccades, and the latency and duration of eye fixation on the desired target. Correlation and regression tests investigated potential relations among participants' changes in multiple variables including task performance, cognitive load, and eye movement variables. The Chi-square and its corresponding *P*-value of 0.05 were used as the criteria to exclude a variable from the model. Values > 0.05 were excluded from the analysis. Exclusion or replacing the unaffected variables did not show to have any significant effect on the pathway models. During the next step, path analysis (standardized regression coefficients and correlation between variables) was used to develop a model to assess how the changes in load indicators affected visual task performance through possible alterations in saccades and/or fixation eye movements. An acceptable model was determined using the criteria of 0.90–1.00 for Comparative Fit Index (CFI) and 0.00–0.06 for Root Mean Square Error of Approximation (RMSEA). The models with the largest CFI and the smallest RMSEA were determined as the final path models selected to analyze the effects of cognitive load on participants' reaction time and response errors submitted for the discrimination task. The IBM SPSS with AMOS Graphics Versions 24 (IBM Corporation, Somers, NY) was used to analyze data.

##  RESULTS

**Figure 1:**
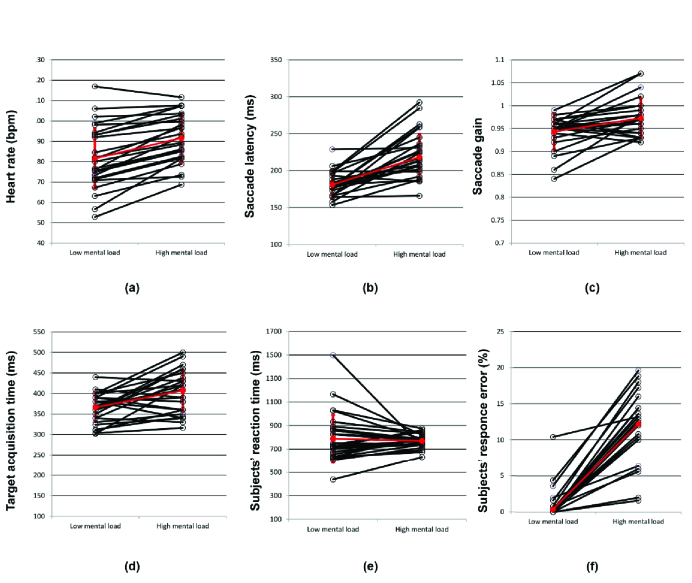
Heart rate (a), saccade latency (b), saccade gain (c), latency of target acquisition; i.e., fixation on target of interest (d), participants' reaction time (e), and response errors (f) in post-saccadic discrimination task under low and high cognitive loads. Black lines represent changes from low to high cognitive load for each participant. Red circles with vertical lines express the mean ± SD under the low and high loads; except for response errors that the red circles express median values. Red diagonal lines represent changes in the means for (a–e) and changes in the medians for (f) from low to high cognitive load.

Both rating scores of mental load and heart rate significantly increased among participants during the task with mental arithmetic when compared to the task without arithmetic (mean difference (SE); for the load score: 15.77 (1.17), *P* < 0.001; for the heart rate: 10.25 (1.17) bpm, *P* < 0.001). It was confirmed that the level of cognitive load significantly increased during the task with arithmetic. Figure 1 shows the changes in heart rate, saccade variables, and task performance from the low to high load within participants. Latencies of saccade and fixation increased significantly during the high load when compared to the low load (for saccade latency: 37.37(32.14) ms, *P* < 0.001; for fixation: 40.86(8.85) ms, *P* < 0.001). The saccade gain was just significantly greater for the high load when compared to the low load (0.029(0.011), *P *= 0.042). The probability of the occurrence of the secondary saccades was less under the high load than under the low load (0.123(0.024), *P* < 0.001). Inaccurate responses submitted on the discrimination task significantly increased under the high load when compared to the low load (10.8% vs 0.97%; Wilcoxon Signed Ranks Test: *P* < 0.001). Fixation duration tended to decrease with the high load, however, the changes in fixation duration and participants' reaction time to the targets were not significant with cognitive load (fixation duration under low load: 391.33(40.36) ms; and under high load: 348.29(11.95) ms; participants' reaction time under low load: 766.49(10.92) ms; and under high load: 785.04 (35.89)).

An inverse correlation was found between changes in fixation duration and response errors from the low to high load (r = –0.54, *P *= 0.004). A significant correlation was found between participants' reaction time to the E targets and latency of fixation on the desired target (r = 0.313, *P *= 0.015). The increase in the response errors submitted for the discrimination task was significantly associated with less probability of the occurrence of secondary saccades (r = –0.401, *P *= 0.001). The correlation between the changes in task performance metrics, that is, reaction time and accuracy of manual responses, from the low to the high load just failed to be significant (r = –0.503, *P* = 0.05).

**Figure 2:**
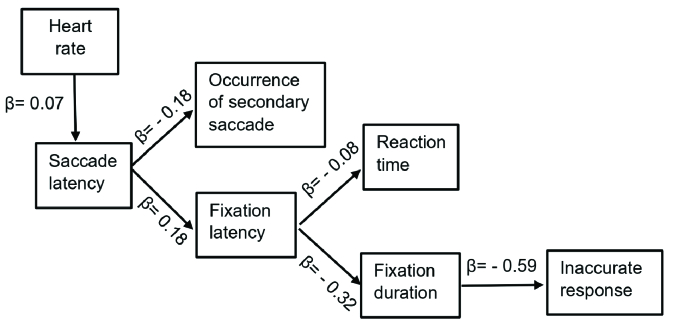
Illustration of final Path Models for the indirect effects of the changes in the cognitive load indicated by heart rate, from low to high load on subjects' reaction time and submission of inaccurate responses.

Path analyses were employed to investigate potential relationships among the task-induced changes in heart rate, MTE score, task performance, and eye movement variables, that is, latency, gain and velocity of saccades, latency and duration of fixation, occurrence of secondary saccades. Figure 2 illustrates the best path models for the effects of task condition on performance metrics including participants' reaction time (Chi-square: 0.098, df = 2, *P *= 0.952, RMSEA: 0.000, NFI = 0.031), and response errors (Chi-square: 6.334, df = 10, *P *= 0.786; RMSEA: 0.000, NFI = 0.737). For both models (the model of reaction time and model of response errors), changes in the task condition was best represented by heart rate and negative effects shown on an individuals' task performance via changes in saccade latency. Changes in heart rate, used as the metric of cognitive load, showed an increasing effect on saccade latency (standardized regression weight [ β = 0.07]). There was an inverse relationship between saccade latency and the probability of the occurrence of secondary saccades ( β = –0.18). The increase in saccade latency was found to delay fixation on the desired target ( β = 0.18) which showed an increasing effect on participants' reaction time ( β = 0.08). The delay in fixation was found to limit fixation duration ( β = –0.32). Shorter duration of fixation was found to contribute in the probability of the submission of inaccurate responses increased on discrimination tasks ( β = –0.59).

##  DISCUSSION

Results obtained by the current study showed cognitive load can indirectly affect an individuals' task performance at the post-saccadic position via changes in eye movements. Increase in saccade latency with high cognitive load can degrade visual task performance indirectly by an increase in the fixation latency and a decrement in the duration of fixation on the desired target.

Consistent with previous studies, our findings showed that cognitive demands affect saccade latency.^[1,5,6,7,8,9,10,11,12,15,16,17]^ Post saccadic visual tasks, such as discrimination, have been found to increase the probability of secondary saccades occurring.^[13]^ However, the probability of secondary saccades developing decreased under the high load possibly because attention allocated to the discrimination task was reduced by the concurrent arithmetic task. Although the nature of secondary saccades is reflexive,^[14]^ the controlled execution of saccades can suppress reflexive saccades under high load conditions.^[5,15]^ In addition, the programming of secondary saccades is done prior to their execution and during the latency of the initial saccades.^[16]^ Like initial saccades, secondary saccades contribute toward the target acquisition time and final eye position.^[17]^ Under cognitive conditions, cost-effective programing of the saccades (trade of between timing and accuracy of saccadic task) might encourage the saccade system to fixate on the desired target more by using a single saccade rather than saccade sequences. Despite changes in the occurrence of secondary saccades with the high load, we did not find any effect of secondary saccades on visual task performance.

Longer saccade latency resulted in the decrease in task performance brought about mainly by an increase in the latency of fixation on the desired target. According to Figure 2, a delay in eye fixation on the target can follow a delay in an individual's reaction time to the target; that is, being slow in reacting to visual stimulus. Our results are consistent with the findings of Wang et al who examined eye movements in a group of patients with involuntary continuous ocular oscillations, whose complaints of delayed visual capture were common (infantile nystagmus).^[18]^ Delayed visual capture can also occur among normally sighted individuals, for example, when drivers report that they looked at a target but either did not see an object or saw it late.^[9]^ “Being slow to see” has been identified clinically as prolonged visual recognition time is predicted as the result of an increase in the latency of fixation; which has been demonstrated by our results that expressed changes in the timing of eye movements and the participant's reaction time during high compared low cognitive loads [Figure 2]. Investigations of eye movements and reaction times in both of these types of patients, those with involuntary continuous ocular oscillations versus normally sighted individuals, concluded that longer times taken to respond to a visual target at post-saccadic positions is mainly as a result of the increased time taken to fixate on a desired target rather than longer saccade latency or the duration of fixation on the target.^[8,19]^ Thus, an increase in recognition time is predicted to follow an increase in the latency of fixation; which has been demonstrated by our results that expressed changes in the timing of eye movements and the participant's reaction time during low to high cognitive loads [Figure 2].

Despite associations between changes in task performance and latency of eye movements, our results did not show any significant differences in the task conditions as it relates to participants' reaction times and fixation durations. Previous investigations have suggested two behavioral profiles for the discrimination tasks with mental arithmetic; those include focusing on either the accuracy or speed of responses at the expense of the other.^[20]^ One possible reason for no significant change in our participants' reaction time can be the perceived task urgency which might result in the participants becoming more concerned about the speed of their performance rather than the accuracy of their responses. Task urgency and accuracy also affect saccade latency albeit via different mechanisms;^[3,21,22]^that is, perceptual urgency – the extent to which the participant feels the time is restricted – can lower the threshold of saccade execution while the other one (accuracy) can affect saccade latency via the information processing system of the brain.^[23]^ It seems that the level of the task urgency perceived by our participants was not large enough to avoid increasing the latency of saccades with mental arithmetic.

According to previous investigations, prolonged visual recognition time is unlikely due to the slow speed of information processing.^[8,19]^ Using a simulated driving task concurrently with phone conversing, Recarte and Nunes measured longer latency of fixation, and more individuals' response errors with no significant change in the participants' reaction time.^[25]^ Consistently, Fadardi and Abel have suggested that efficiency of information processing, despite no significant change in its duration, gives rise to resolving target details including both visual inputs and processing of the information during fixation.^[8]^ Previous results have suggested that performance was not degraded due to the time restriction to perform a visual task but resulted from poor information processing.^[8,25]^ According to our results, a decrement in fixation duration predicted more response errors in an individual's task performance [Figure 2]. In other words, a subject who showed longer fixation duration is expected to submit a correct response to a post-saccadic visual task. Our results are consistent with previous findings by Tsai et al who used auditory arithmetic evaluations concurrently with a simulated driving task.^[26]^ Their results showed that although fixation duration did not change across cognitive levels, they could predict upcoming errors when it decreased just before an error was made in the arithmetic task.^[26]^ The results obtained through the scene viewing studies have shown that information extracted during fixation affects the onset timing of the subsequent saccade essentially terminating that fixation.^[27]^ Another study showed that increased cognitive level of a scene viewing task resulted in longer duration of fixation.^[27]^The task design and the comparisons within subjects that were used in the current study make it unlikely for the target features to affect our results obtained for fixation duration. The efficiency of the information processing can be reflected in the participants' task performance in terms of both the speed and accuracy of the responses.^[24]^ According to our results, it seems that providing a correct response to the visual target requires longer time in the presence of a limited capacity of information processing or the quality of processing being degraded despite the presence of healthy oculomotor systems.

The current study showed that varied levels of cognitive load changed individuals' response errors and reaction times indirectly through changes in saccade latency. Increased saccade latency predicts longer time requiring for getting the eyes on the desired target. If the allocated time duration between the onset of visual onset and providing response to the target is restricted, a delay in eye fixation on the target can further limit fixation duration before the subject submits the response to the visual target. Our data showed that saccade latency can be used as an indicator of cognitive load, but also flags degraded efficiency of information processing which can demand longer fixation duration to protect accuracy of the subject's response to the visual target. Therefore, latencies of saccade and fixation can be used to monitor forecasted performance especially when both time and accuracy matter, for example, when driving.

##  Ethical approval

All procedures performed in studies involving human participants were in accordance with the ethical standards of the institutional and/or national research committee and with the 1964 Helsinki declaration and its later amendments or comparable ethical standards.

##  Financial Support and Sponsorship 

This work was supported by the University of Melbourne.

##  Conflicts of Interest

No conflicting relationship exists for any author.
